# Expression of the neuron-specific protein CHD5 is an independent marker of outcome in neuroblastoma

**DOI:** 10.1186/1476-4598-9-277

**Published:** 2010-10-15

**Authors:** Idoia Garcia, Gemma Mayol, Eva Rodríguez, Mariona Suñol, Timothy R Gershon, José Ríos, Nai-Kong V Cheung, Mark W Kieran, Rani E George, Antonio R Perez-Atayde, Carla Casala, Patricia Galván, Carmen de Torres, Jaume Mora, Cinzia Lavarino

**Affiliations:** 1 Developmental Tumor Biology Laboratory, Hospital Sant Joan de Déu, Fundación Sant Joan de Déu, Barcelona, Spain; 2 Department of Pathology, Hospital Sant Joan de Déu, Barcelona, Spain; 3 Department of Neurology, University of North Carolina, Chapel Hill, USA; 4 Laboratory of Biostatistics & Epidemiology, Universitat Autònoma de Barcelona; Clinical Pharmacology Service, IDIBAPS, Hospital Clinic, Barcelona, Spain; 5 Department of Pediatrics, Memorial Sloan-Kettering Cancer Centre, New York, USA; 6 Division of Pediatric Hematology/Oncology, Dana-Farber Cancer Institute and Children's Hospital of Boston, USA; 7 Department of Pathology, Children's Hospital of Boston, USA

## Abstract

**Background:**

The chromodomain, helicase DNA-binding protein 5 (CHD5) is a potential tumor suppressor gene located on chromosome 1p36, a region recurrently deleted in high risk neuroblastoma (NB). Previous data have shown that *CHD5 *mRNA is present in normal neural tissues and in low risk NB, nevertheless, the distribution of CHD5 protein has not been explored. The aim of this study was to investigate CHD5 protein expression as an immunohistochemical marker of outcome in NB. With this purpose, CHD5 protein expression was analyzed in normal neural tissues and neuroblastic tumors (NTs). *CHD5 *gene and protein expression was reexamined after induction chemotherapy in a subset of high risk tumors to identify potential changes reflecting tumor response.

**Results:**

We provide evidence that CHD5 is a neuron-specific protein, absent in glial cells, with diverse expression amongst neuron types. Within NTs, CHD5 immunoreactivity was found restricted to differentiating neuroblasts and ganglion-like cells, and absent in undifferentiated neuroblasts and stromal Schwann cells. Correlation between protein and mRNA levels was found, suggesting transcriptional regulation of *CHD5*. An immunohistochemical analysis of 90 primary NTs highlighted a strong association of CHD5 expression with favorable prognostic variables (age at diagnosis <12 months, low clinical stage, and favorable histology; P < 0.001 for all), overall survival (OS) (P < 0.001) and event-free survival (EFS) (P < 0.001). Multivariate analysis showed that CHD5 prognostic value is independent of other clinical and biologically relevant parameters, and could therefore represent a marker of outcome in NB that can be tested by conventional immunohistochemistry. The prognostic value of CHD5 was confirmed in an independent, blinded set of 32 NB tumors (P < 0.001).

Reactivation of *CHD5 *expression after induction chemotherapy was observed mainly in those high risk tumors with induced tumor cell differentiation features. Remarkably, these NB tumors showed good clinical response and prolonged patient survival.

**Conclusions:**

The neuron-specific protein CHD5 may represent a marker of outcome in NB that can be tested by conventional immunohistochemistry. Re-establishment of CHD5 expression induced by chemotherapy could be a surrogate marker of treatment response.

## Introduction

Neuroblastic tumors (NTs) are embryonal cancers arising from neural crest derived sympathetic nervous system precursors. These neoplasms are the most common extracranial solid tumors in childhood and account for approximately 15% of all pediatric oncology deaths [1].

Neuroblastoma (NB), the most undifferentiated form of NTs, embodies a heterogeneous spectrum of diseases whereby patients with similar clinicopathological features exhibit radically different outcomes ranging from spontaneous regression to inexorable progression. Since treatment strategies vary from a "watchful waiting" approach to multimodal intensive regimens, precise risk assessment is critical for therapeutic decisions. Various combinations of prognostic markers have been used with success for risk group distinction, including clinical, histologic and genetic factors, yet there remain cases where established indicators of aggressiveness have demonstrated limited clinical utility. Additional parameters are therefore needed for a more precise identification and therapeutic targeting of high risk NB patients.

There is an apparent link between NB aggressiveness and specific genetic aberrations. One of the most recurrent genetic alterations described is the deletion of the short arm of chromosome 1 found in approximately 35% of NB [2]. The high incidence of chromosome 1p deletion in human cancer [2], with 1p36 deletion being the most common alteration [3], has led to an extensive search for 1p36 tumor suppressor genes. Recent findings have identified the *CHD5 *gene as a candidate tumor suppressor [4,5] mapping to the smallest region of deletion (SRD) described in NB, 1p36.31 [6]. Evidence supporting *CHD5 *as a tumor suppressor is the recently reported strong promoter methylation and transcriptional silencing of the remaining allele in 1p deleted NB cell lines [5]. Nevertheless, low or absent *CHD5 *expression levels have been found in NB cell lines lacking promoter methylation [7], 1p deletion, or inactivating mutations [6], suggesting other mechanisms by which *CHD5 *expression may be inhibited.

CHD5 is one of the nine members of the chromodomain helicase DNA-binding (CHD) family of enzymes that belong to the ATP-dependent chromatin remodeling protein SNF2 superfamily [8]. CHD protein structure is characterized by two N-terminal chromodomains and a SNF2-like ATPase central domain that defines the chromodomain remodeling proteins [9,10]. The members of this evolutionarily conserved class of proteins play a critical role in organizing the chromatin structure and accordingly, in chromatin based transcriptional regulation of genes.

The aberrant expression of some of the CHD genes has been associated with human disease processes like CHARGE syndrome, Hodgkin's lymphoma or dermatomyositis [8]. *CHD5 *mRNA expression, restricted to neuronal-derived tissues and the adrenal gland in normal tissues [10], is basically absent in NB primary tumors with high risk features, *MYCN *amplification, advanced stage and 1p monosomy [5].

The distribution of CHD5 protein in NTs and normal neural tissues has not been explored. Like neural tissue, NTs consist of two main cell populations, neuroblastic cells and Schwann-like cells. The malignant potential of these tumors is inherently dependent on the proportion of immature neuroblastic cells and the abundance of Schwann cell stromal component, Schwannian stroma-poor undifferentiated NB being the most malignant. CHD5 expression remains to be investigated in these two cell populations. In the present study, we analyzed by immunohistochemistry normal neural derived tissues and NTs to visualize CHD5 protein distribution within the different cell populations. Because impaired *CHD5 *expression is associated with high risk NB tumors, we asked whether CHD5 protein expression might serve as an immunohistochemical marker of outcome in NB. It is known that gene expression pattern can change with treatment, for this reason, *CHD5 *gene and protein expression was re-examined after induction treatment in a set of paired cases.

## Material and Methods

### Patients and tumor samples

A total of 90 primary tumor specimens (63 NB, 14 ganglioneuroblastomas (GNB) and 13 ganglioneuromas (GN)) (Additional file 1) were obtained at diagnosis from two institutions (Hospital Sant Joan de Déu (HSJD) of Barcelona and Memorial Sloan-Kettering Cancer Center (MSKCC) of New York) together with 12 high risk NB cases with available paired diagnostic and post-chemotherapy tumor specimens. An independent set of 32 NB tumors was obtained from Children's Hospital of Boston and Dana-Farber Cancer Institute (CHB/DFCI) for data validation analysis. Non-tumor samples (fetal brain, adult cerebral cortex, adult cerebellum, adrenal gland, bone marrow, spinal cord and sympathetic ganglion) were also included in this study.

NB risk assessment was defined by the International Neuroblastoma Staging System (INSS) [11]. NB stages 1, 2, 3 (*MYCN *non-amplified) and 4s were uniformly treated without use of cytotoxic therapy, when possible. Stage 4 and stage 3 *MYCN *amplified NB patients were treated according to N5, N6 or N7 protocols. This study was approved by the Institutional Review Boards and informed consent was obtained before collection of samples.

Tumors were assessed by a pathologist (M.S.), only tumors with >70% viable tumor cell content were included in the study.

Seven NB cell lines (LA-N-1, SKNSH-SY5Y, SK-N-Be(2)C, SKNSH-EP1, SK-N-JD, SK-N-LP and SK-N-AS) were used in this study. NB cell lines were cultured in RPMI-1640 supplemented with 10% fetal bovine serum (FBS), 2 mM L-glutamine and penicillin (100 U/ml) and streptomycin (100 μg/ml) (GIBCO, Invitrogen, US) at 37°C in 5% CO_2 _atmosphere.

### *In vivo *study

NB cell lines SK-N-JD, SK-N-LP and SK-N-AS were harvested and resuspended in phosphate buffered saline (PBS) solution and BD Matrigel Basement Membrane Matrix (BD Biosciences, US). One hundred microliters of cell suspension containing 8 × 10^6 ^cells were subcutaneously inoculated into the right flank of six-week old CD-1 Nude (nu/nu) mice (Charles River Laboratories, Europe). Mice were killed when NB cell lines developed tumors that exceeded 1.5 cm^3^. Tumors were removed surgically, fixed in 10% formalin and embedded in paraffin for histological examination.

### Immunohistochemistry

Immunohistochemical (IHC) analysis was performed on formalin-fixed, paraffin-embedded (FFPE) tissues using rabbit-polyclonal anti-CHD5 antibody (Strategic Diagnostics, DE) at a 1:1000 dilution for 1 hour; mouse-polyclonal anti-Neurofilament protein, 68kD (NF68) antibody (Zymed, US) 1:300 dilution, 1 hour and mouse-polyclonal anti-Glial fibrillary acidic protein (GFAP) antibody (Novocastra, UK) 1:200 dilution, 2 min. Two different anti-CHD5 antibody batches (T00251-A1 and T00251-A02, Strategic Diagnostics, DE) have been tested in this study. Normal human brain was used as positive control.

Slides were examined by a pathologist (M.S.) using an Olympus BX41 light microscopy to assess staining and score both percentage of positive cells and staining intensity (0, negative; 1, weak; 2, strong and 3, very intense staining). Integer values were assigned to the proportion of positive cells (<25% = 1; 25-75 = 2; >75% = 3). Intensity and positive cell values were multiplied to provide a single score for each case.

*Double fluorescent immunostaining: *Paraformaldehyde (4%, pH 7.4) fixed cryosections, blocked with bovine serum albumin (BSA) 1% for 1 hour, were incubated overnight at 4°C with a rabbit-polyclonal anti-CHD5 antibody (H-185) (Santa Cruz, US) at 1:1000 dilution, followed by anti-rabbit IgG Cy3-conjugated antibody, (Sigma, US) 1:400 dilution for 45 min. Sections were subsequently incubated with anti-NF68 antibody (1:300 dilution) 1 hour or anti-GFAP antibody (1:200 dilution) 2 min, and stained with anti-mouse IgG FITC-conjugated antibody (Sigma, US) 1:700 dilution, 45 min. Nuclei were counterstained with 4'6-diamino-2-phenylindole (DAPI) (Sigma, US), 1:5000 dilution, 5 min.

Paraformaldehyde fixed bone marrow aggregates were incubated with anti-GD2 antibody (BD Biosciences, US) 1:800 dilution 1 hour and stained with anti-mouse IgG-FITC antibody at 1:700 dilution, 45 min, or with anti-CHD5 antibody as described above.

Immunoreactivity was evaluated with a Leica epifluorescence DM5000B microscope (Leica Microsystems, US).

### Western blot analysis

Proteins were extracted from cell lines and homogenized tissue in lysis buffer (20 mM Tris pH 8.8, 80 mM NaCl, 1% NP-40 and protease inhibitors). Protein concentrations were quantified using the Bradford method (Bio-Rad laboratories, US) and 30 μg of protein were resolved on an 8% SDS-PAGE. Membranes were incubated with polyclonal anti-CHD5 antibody (1:2000; Strategic Diagnostics, DE) and monoclonal anti β-actin antibody (1:5000; Sigma, US) and detected with donkey anti-rabbit IgG HRP-conjugated antibody (1:2500; Affinity BioReagents, Inc., US) and goat anti-mouse IgG HRP-conjugated antibody (1:5000; Sigma, US) respectively. Antibody conjugates were visualized by enhanced chemiluminescence (ECL, Amersham Life Science, US).

### RNA isolation and cDNA synthesis

Total RNA was isolated from snap frozen samples and cell lines using Tri Reagent (Sigma, US), following manufacturers' protocols. cDNA was synthesized from 1 μg total RNA using random primers and M-MLV reverse transcriptase (Promega, US) as previously described [12].

### Quantitative Real-time Polymerase Chain Reaction (qRT-PCR)

Quantification of transcript levels, using the ΔΔC_T _relative quantification method, were performed on an ABI Prism 7000 Sequence Detection System with TaqMan^® ^Assay-on-Demand Gene Expression products (Applied Biosystems, US), as previously reported [12].

### Statistical analysis

Comparisons between immunohistochemical results were performed by means of the log-rank test. qRT-PCR transcript levels were normalized by z-score transformation to enable a correlation analysis with the immunostaining score values. Correspondence between immunoreactivity and mRNA expression levels within the same samples was examined using the Spearman's correlation coefficient analysis. Statistical analyses for qualitative variables were performed by means of the Fisher's exact test and U Mann-Whitney test for quantitative or ordinal variables. Overall survival (OS) and event-free survival (EFS) probabilities were estimated using the Kaplan-Meier method. Multivariate Cox regression models were used to examine the prognostic significance of CHD5, INSS stage, age at diagnosis, *MYCN *status and 1p LOH. Each variable consisted of two groups: "INSS stage" consisted of: (1) ST1, 2, 3 and 4s, and (2) ST4; "age" (at diagnosis): (1) ≤ 12 months (2) > 12 months; "*MYCN*": (1) *MYCN *non-amplified (2) *MYCN *amplified; "LOH": (1) no LOH (2) LOH. Predictive Positive and Negative Values (PPV and NPV) were used for a descriptive comparison between CHD5 expression and *MYCN *and 1p LOH. All reported P-values are two-sided. P-values ≤0.05 were considered statistically significant. Statistical analysis was performed with SPSS 15.0 package (SPSS, Chicago, IL).

## Results

### CHD5 protein expression in normal neural tissues is restricted to neuronal cells

**Figure 1:**
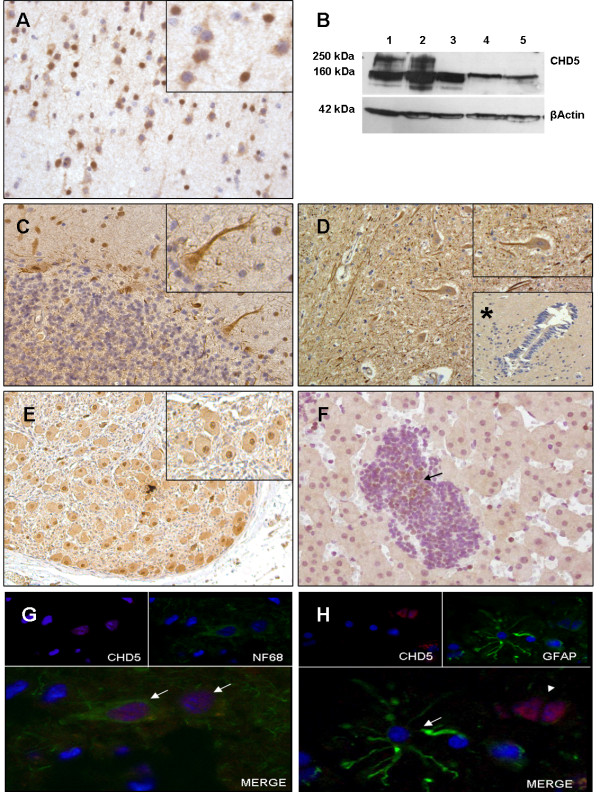
**CHD5 protein expression in normal human neural tissues. **CHD5 immunostaining in **(A)** normal neural tissue, cerebral cortex (100x), box: pyramidal and interneuron CHD5 staining (400x); **(B)** expression of CHD5 protein detected by immunoblotting in (1) brain cortex total protein, (2) brain cortex nuclear protein fraction, (3) brain cortex cytoplasmatic fraction, (4) LA-N-1 NB cell line total protein and (5) LA-N-1 nuclear fraction; CHD5 predicted molecular weight of 250-260 kDa is based on the amino acid composition (ref. 5); all analyzed samples displayed a 150-160 kDa size band, not yet characterized; **(C)** Cerebellum (100x), box: Purkinje cell CHD5 staining (400x); **(D)** Spinal cord (100x), box: Motoneuron CHD5 staining (400x), box*: ependymal cells lining canal spinal cord (100x); **(E)** Sympathetic ganglia (100x), box: Ganglion cell CHD5 staining (400x); **(F)** immature neuroblast aggregates within fetal adrenal gland (200x), (arrow) CHD5 immunopositive neuroblasts; **(G-H)** double fluorescent immunostaining, (G, arrow) NF68^+^/CHD5^+ ^neuron; (H, arrow) GFAP^+^/CHD5^- ^astrocyte, (H, arrow head) GFAP^-^/CHD5^+ ^neuron.

In normal human neural tissue sections (brain cortex, cerebellum, spinal cord and sympathetic ganglion), CHD5 immunoreactivity was found restricted to neurons, whereas glial cells were consistently negative (Figure 1A, C, D and 1E). CHD5 expression pattern was confirmed by immunostaining with neuronal (NF68) and glial (GFAP) cell markers. Frozen brain sections analyzed by double immunofluorescence showed co-localization of CHD5 and NF68 in neurons. No CHD5 protein expression was observed in GFAP positive glial cells (Figure 1G and 1H).

Intensity and intracellular localization of CHD5 staining in the cerebral cortex varied among neuron types but did not exhibit a layer-related expression (Figure 1A). Nuclear labeling was intense in morphologically small neurons with scarce cytoplasm present in all cortical layers identified by size and location as interneurons. Larger neurons with triangular shaped soma, including pyramidal neurons present in cortical layers III, IV and V, exhibited essentially negative or lower intensity of nuclear staining and diffuse cytoplasm reactivity (Figure 1A). In the cerebellum, Purkinje cells and deep nuclei neurons exhibited intense nuclear and diffuse cytoplasm staining. Cerebellar granular layer neurons lacked immunoreactivity (Figure 1C).

Spinal cord specimens were characterized by intense positive neuron processes, predominantly located in the external white matter, and large motoneuron cell bodies with positive cytoplasm and mostly negative nuclear staining (Figure 1D). All glial cells, including the ependymal cells lining the central canal of the spinal cord, were negative for CHD5 expression (Figure 1D*). In the sympathetic ganglia, neuron cell bodies showed intense nuclear and diffuse cytoplasm reactivity, while the stromal cell component was found negative for CHD5 (Figure 1E).

Adrenal gland specimens exhibited weak CHD5 expression, mainly in the nucleus of the medullary cells. Neuroblastic aggregates found in fetal adrenal glands (19-20 weeks) were essentially negative, although few intermixed positive cells were identified in larger neuroblastic islets (Figure 1F).

CHD5 expression was evaluated in brain cortex specimens and in NB cell lines by immunoblot analysis. CHD5 protein (250-260 kDa) was detected only in brain cortex specimens, both in the total protein extract and in the nuclear fraction. No CHD5 protein was detected in the cytoplasmic fraction of all the analyzed specimens or in NB cell lines (Figure 1B).

These results identify CHD5 as a neuron-specific protein, absent in glial cells, with a diverse expression pattern amongst neuron types. Human immature neuroblastic aggregates in the developing adrenal gland are mostly negative for CHD5.

### CHD5 protein is expressed in the neuroblastic component of low clinical risk NTs

CHD5 immuno-localization was investigated in a total of 90 primary NTs (63 NB including 24 stage 4, 8 stage 4s and 31 loco-regional NB; 14 GNB and 13 GN) (Table 1).

**Table 1:** CHD5 inmunostaining in Neuroblastic tumors.

		Percentage of CHD5 immunopositive neuroblastic cells		
		
	n	<25%	25-75%	>75%
**St 1,2,3**	31	10/31 (32.2%)	9/31 (29%)	12/31 (38.7%)
**St 4**	24	19/24 (79.1%)	4/24 (16.6%)	1/24 (4.1%)
**St 4s**	8	--	--	8/8 (100%)
**GNB**	14	14/14* (100%)	--	14/14** (100%)
**GN**	13	--	--	13/13 (100%)

**Total**	90	29	13	48

Percentage of CHD5 positive (nuclear staining) tumor cells within each neuroblastic tumor group evaluated using predetermined cutoff values (<25%; 25-75%; >75%). Detailed data regarding percentage of positive tumor cells and staining intensity are reported in Additional file 1. For GNB tumors, the undifferentiated neuroblastic (*) and the ganglionar (**) cell populations were scored separately. GNB = ganglioneuroblastoma, GN = Ganglioneuroma.

**Figure 2:**
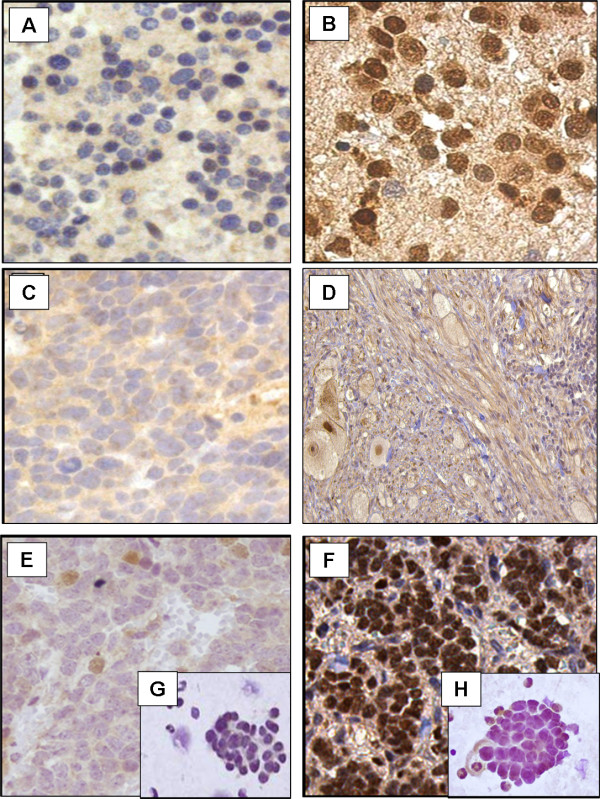
**CHD5 protein expression in neuroblastic tumors. **CHD5 immunostaining in **(A)** stage 4 undifferentiated NB (400x); **(B)** loco-regional differentiating NB (400x); **(C)** stage 3, *MYCN *amplified, undifferentiated NB (400x); **(D)** Ganglioneuroblastoma (100x); **(E)** stage 4 (200x) and **(F)** stage 4s primary tumor (200x) with bone marrow neuroblast aggregates **(G-H)**, respectively (400x).

Stage 4 NB cases, all histologically undifferentiated high risk NB, appeared predominantly (20/24) negative or with <25% neuroblastic cells with faint CHD5 nuclear reactivity (Figure 2A and 2E; Table 1, Additional file 1). Only 3/24 undifferentiated NB tumors exhibited weak nuclear reactivity in 25-75% of cells, and one had intense nuclear staining in >75% of tumor cells. In contrast, stage 4s NB, histologically undifferentiated low risk tumors, showed consistently (8/8) very intense CHD5 nuclear positivity in >75%, generally >90% of the neuroblasts (Figure 2F, Table 1, Additional file 1). This clinically low risk NB is, nevertheless, a highly proliferative metastatic tumor. Thus, for 2 stage 4s NB tumors, CHD5 expression was also evaluated in the liver and bone marrow metastases. Intense CHD5 immunopositivity, equivalent to the primary tumor, was observed in >75% neuroblasts disseminated in the liver. Intriguingly, bone marrow neuroblastic aggregates, identified using an antibody against the ganglioside GD2 ubiquitously expressed in NB (data not shown), lacked CHD5 immunoreactivity (Figure 2H), similar to stage 4 bone marrow smears (Figure 2G).

Loco-regional tumors (stage 1, 2, and 3) displayed more heterogeneous expression patterns (Figure 2B and 2C; Additional file 1), with staining values being highest in differentiating NB, where intense nuclear staining was observed in >75% of neuroblastic cells (13/32) (Figure 2B; Additional file 1), and lowest in stage 3 *MYCN *amplified NB composed mainly of undifferentiated neuroblasts with undetectable immunoreactivity, similar to stage 4 NB cases (Figure 2C, Table 1, Additional file 1).

GNB (14/14) and GN (13/13) tumors exhibited ganglion-like cells with intense nuclear and diffuse cytoplasm staining. Absence of nuclear staining and feeble cytoplasmic reactivity was observed in Schwann-like cells (Figure 2D; Additional file 1). The undifferentiated neuroblastic component of GNB lacked CHD5 staining (Table 1, Additional file 1).

The described immunohistochemical assays were performed using two different batches of the anti-CHD5 antibody (T00251-A1 and T00251-A02). Both batches performed consistently across many repeats, further supporting the validity of our results (Additional file 2A). The specificity of the anti-CHD5 antibody was further validated on mouse xenografts of human NB cell lines (SK-N-JD, SK-N-LP and SK-N-AS). All the xenografts were found to be negative for CHD5 staining (Additional file 2B).

Altogether, CHD5 protein was expressed in the nucleus of neuroblastic cells of clinical low risk NTs. In stage 4s NB, CHD5 negative neuroblast bone marrow metastasis imply the existence of intratumoral clones with CHD5 differential expression in an otherwise histologically homogeneous tumor subtype.

### *CHD5 *transcript levels are associated with protein expression

CHD5 protein expression was contrasted with gene transcript levels. Quantification of *CHD5 *mRNA in non-tumoral frozen tissue samples using qRT-PCR identified high expression in fetal brain and adult cerebral cortex, as reported previously [10]. Normal bone marrow specimens lacked *CHD5 *expression.

*CHD5 *mRNA levels were analyzed for 84 primary NTs obtained at diagnosis (23 stage 4; 7 stage 4s; 34 loco-regional NB; 9 GNB and 11 GN); 55 of these tumors were also analyzed by immunohistochemistry.

High risk undifferentiated NB tumors, stage 4 and stage 3 *MYCN*-amplified NB displayed significantly lower mRNA expression levels than stage 1, 2, 3 (P < 0.001) and stage 4s NB (P = 0.001) (Additional file 3). The highest mean expression values, similar to normal fetal brain, were found for stage 4s NB. GN specimens displayed consistently low *CHD5 *transcript levels, whereas, GNB tumors were characterized by highly variable expression attributable to the presence of *CHD5 *negative component, Schwann-like stroma and undifferentiated neuroblasts, besides the positive ganglion-like cells that compose these tumors.

Correlation between CHD5 immunoreactivity and mRNA expression levels within the same samples was examined in a set of 34 consecutive NB tumors. Immunohistochemical and qRT-PCR analyses were carried out on the same portion of the tumor specimen, with similar cell composition and a high tumor cell content (>70% as recommended for PCR studies). CHD5 nuclear immunoreactivity was assigned a staining score (Additional file 1) and gene expression values were z-score transformed. A significant correlation was observed between mRNA and protein levels (Spearman's rho = 0.774; P < 0.001), low CHD5 protein scores were consistently associated with low mRNA levels (negative z-score values), and high IHC scores with high mRNA expression (positive z-score values) (Additional file 4). Interestingly, very intense nuclear staining displayed by low risk tumors, mostly stage 4s and infant stage 1 NB, was not associated with the highest transcript levels (Additional file 4, cases # 1-6, 30, 31 and 33).

These results reveal a correspondence between CHD5 protein and mRNA expression, suggesting a potential regulation of *CHD5 *expression at the transcriptional level.

### CHD5 protein expression is associated with patient outcome in NB

CHD5 nuclear immunoreactivity was assigned a staining score (Additional file 1) and compared to clinical and biological variables currently used for NB risk classification. High CHD5 staining values were found to be significantly associated with INSS stages 1, 2, 3 (*MYCN *non-amplified) and 4s NB (n = 63), age at diagnosis <12 m (n = 63) and favorable tumor histology (n = 63); P < 0.001 for all the tested variables.

**Figure 3:**
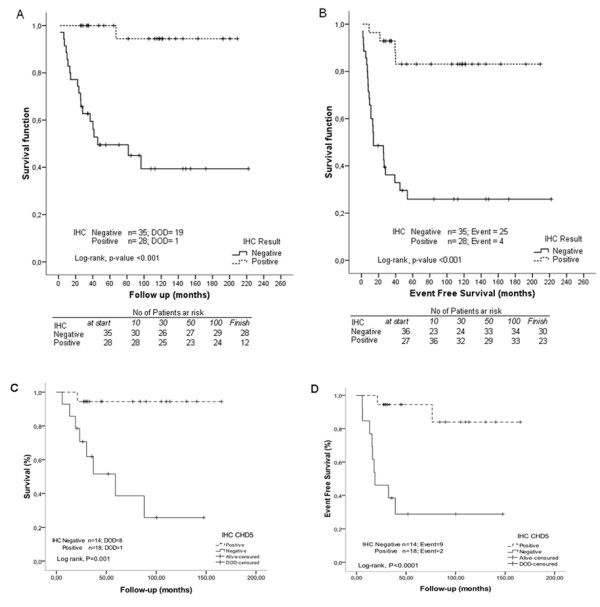
**Expression of CHD5 protein is prognostic for neuroblastoma patients**. Kaplan-Meier analysis documenting increased overall survival and event-free survival of neuroblastoma patients with tumors that have high CHD5 immunoreactivity *versus *patients with tumors that have low CHD5 expression, **(A) **overall survival (P < 0.001) and **(B) **event-free survival (P < 0.001). The analysis was performed with all 63 NB tumors, including all stages (stage 1, 2, 3, 4s and 4). Independent validation performed on 32 primary NB samples **(C) **overall survival (P = 0.001) and **(D) **event-free survival (P < 0.0001). DOD = dead of disease.

To assess whether CHD5 expression was associated with patient outcome, immunoreactivity scores were compared to overall survival (OS) and event-free survival (EFS) for all 63 NB tumors. The median score value was used as a cut-off to define high (>2) and low (≤2) CHD5 expressing NB tumors. High CHD5 expression was found to be significantly associated with a better OS (log-rank test P < 0.001) and EFS (log-rank test P < 0.001) (Figure 3A and 3B). Furthermore, using this cut-off, Cox multivariate analysis showed that expression of CHD5 protein predicted OS and EFS independently of INSS stage, patient age, amplification of *MYCN *and 1p LOH (Table 2; Additional file 5). Specifically, CHD5 IHC was the only variable that remained statistically significantly associated with event-free survival in both the univariate and multivariate analyses (Table 2; Additional file 5). CHD5 IHC remained statistically significantly associated with overall survival, except when 1p LOH was included in the multivariate analysis, owing to the strong association of *CHD5 *expression with chromosome 1p status (Table 2; Additional file 5). The Predictive Value of CHD5 staining was evaluated and compared to *MYCN *and 1p LOH (Table 3). CHD5 expression showed the highest Negative Predictive Value (NPV) for overall survival status (96.4%) and event free survival (85.7%). *MYCN *status and 1p LOH showed a NPV of 81.6% and 78.9%, respectively, for the overall survival status, and of 65.3% and 63.2%, respectively, for the event free survival. The Positive Predictive Value (PPV) of CHD5 expression (overall survival status: 54.3%; event free survival: 71.4%), i.e. proportion of events or deaths in patients with low CHD5 expressing tumors, was intermediate between *MYCN *(66.7% and 77.8%, respectively) and 1p LOH (46.7% and 60%, respectively) values. CHD5 IHC showed high sensitivity and accuracy rate for the prediction of OS (95% and 73%, respectively) and EFS (86.20% and 77.80%) (Table 4).

**Table 2:** Cox regression analysis.

Overall Survival						
**Variable**	**HR and 95%CI**	**p-value**	**Variable**	**HR and 95%CI**	**p-value**	**Interaction p-value**^**&**^

**CHD5 IHC**	21.28 (2.84-159.39)	**0.003**				

			**CHD5 IHC**	18.67 (2.27-153.88)	**0.007**	0.951
**INSS**	4.15 (1.63-10.58)	0.003	**INSS**	1.24 (0.46-3.29)	0.673	

			**CHD5 IHC**	15.17 (1.89-121.73)	**0.011**	0.931
**Age (>12 m)**	5.56 (1.61-19.19)	0.007	**Age (>12 m)**	2.14 (0.59-7.75)	0.249	

			**CHD5 IHC**	12.29 (1.56-96.49)	**0.017**	0.952
*MYCN *(*)	14.27 (4.28-47.58)	< 0.001	*MYCN*(*)	8.08 (2.43-26.93)	0.001	
			**CHD5 IHC**	14.33 (1.84-111.48)	**0.011**	0.946
LOH(**)	2.88 (1.04-7.96)	0.042	LOH(**)	1.75 (0.63-4.89)	0.287	

**Event Free Survival**						

**Variable**	**HR and 95%CI**	**p-value**	**Variable**	**HR and 95%CI**	**p-value**	**Interaction p-value**^**&**^

**CHD5 IHC**	8.14 (2.82-23.5)	<0.001				

			**CHD5 IHC**	7.01 (2.09 to 23.51)	0.002	0.939
**INSS**	3.7 (1.73-7.88)	0.001	**INSS**	1.26 (0.53 to 2.97)	0.605	

			**CHD5 IHC**	7.04 (2.24 to 22.09)	0.001	0.638
**Age (>12 m)**	3.13 (1.33-7.39)	0.009	**Age (>12 m)**	1.36 (0.54 to 3.44)	0.515	

			**CHD5 IHC**	5.97 (1.98 to 17.98)	0.001	0.933
*MYCN *(*)	4.58 (1.84-11.42)	0.001	***MYCN***	2.72 (1.07 to 6.88)	0.035	

			**CHD5 IHC**	6.02 (1.98 to 18.34)	0.002	0.492
LOH(**)	2.12 (0.92-4.91)	0.079	**LOH**	1.24 (0.52 to 2.95)	0.622	

Cox regression analysis of the NB cohort using CHD5 protein expression and clinical and biologically relevant variables (INSS stage, patient age at diagnosis, *MYCN *amplification status and chromosome 1p LOH). Expression of CHD5 was statistically significantly associated with overall survival and event-free survival in both the univariate and multivariate analyses. All NB patients (n = 63) where included in the study except for the studies with *MYCN *amplification (n = 58) and 1p LOH (n = 53), due to the undetermined status in some patients. IHC = Immunohistochemical analysis; INSS = International Neuroblastoma Staging System; HR = hazard ratio; CI = confidence interval. P-values are two sided. (^**&**^**) **Interaction P-values obtained from Cox regression model: IHC + Co-factor+IHC*Co-factor. (*) data available for 58 patients; (**) data available for 53 patients.

**Table 3:** Analysis of the Predictive Value was performed for a descriptive comparison between CHD5 expression and *MYCN *and 1p LOH.

		Overall Survival				Event Free Survival			
		
		Alive	Dead	PPV	NPV	No event	Event	PPV	NPV
**CHD5 IHC**	**High**	27 (62.8%)	1 (5%)	54.30%	96.40%	24 (70.6%)	4 (13.8%)	71.40%	85.70%
	**Low**	16 (37.2%)	19 (95%)			10 (29.4%)	25 (86.2%)		

***MYCN***	**Non amplified**	40 (93%)	9 (60%)	66.70%	81.60%	32 (94.1%)	17 (70.8%)	77.80%	65.30%
	**Amplified**	3 (7%)	6 (40%)			2 (5.9%)	7 (29.2%)		

**1p LOH**	**No**	30 (78.9%)	8 (53.3%)	46.70%	78.90%	24 (80%)	14 (60.9%)	60%	63.20%
	**LOH**	8 (21.1%)	7 (46.7%)			6 (20%)	9 (39.1%)		

**Table 4:** Comparison of sensitivity, specificity and accuracy rate between CHD5 expression, *MYCN *status and 1p LOH.

		Overall Survival			Event Free Survival	
	
	Sens	Specif	Accurancy	Sens	Specif	Accurancy
**CHD5 IHC**	95.00%	62.80%	73.00%	86.20%	70.60%	77.80%

***MYCN***	40.00%	93.00%	79.30%	29.20%	94.10%	67.20%

**1p LOH**	46.70%	78.90%	69.80%	39.10%	80.00%	62.30%

						

The prognostic value of CHD5 expression was validated on an independent, blinded set of 32 FFPE primary NB tumors of patients diagnosed and treated at the Children's Hospital of Boston (n = 21) and HSJD of Barcelona (n = 11). Kaplan-Meier analysis and a log-rank test showed a statistically significant difference in OS (log-rank test P = 0.001) and EFS (log-rank test P < 0.0001) between patients with high and low CHD5 expression scores (Figure 3C and 3D). Tumors with high IHC scores were associated with longer survival (mean 73 months) in comparison with low expressing tumors (mean 46 months).

These results suggest that CHD5 protein expression is a potential prognostic marker of outcome in NB patients.

### *CHD5 *expression reactivation is associated with tumor response to induction therapy

**Figure 4:**
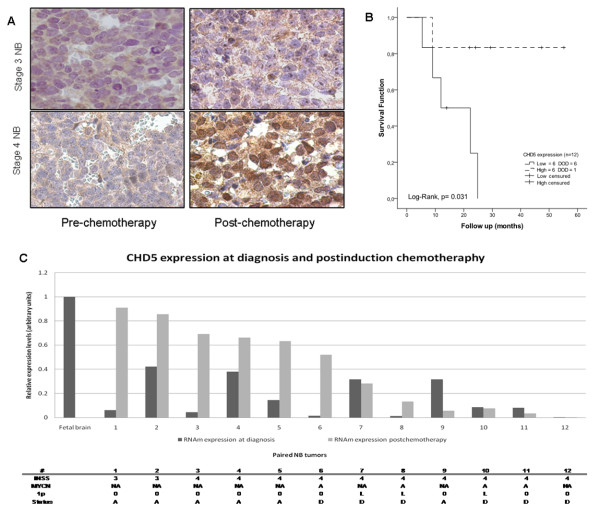
**Chemotherapy-induced expression of *CHD5 *in 12 high risk NB tumors.** (A) CHD5 immunoreactivity pre- and post-chemotherapy (400x); (B) Kaplan-Meier analysis for OS was performed using the mean between pre-treatment and post-treatment gene expression levels as cut-off to divide tumors which reactivate *CHD5 *and tumors that maintain low expression levels; (C) *CHD5 *transcript levels quantified by qRT-PCR: *CHD5 *expression levels (Black) at diagnosis, (Grey) post-chemotherapy. Fetal brain *CHD5 *expression represents normal neural tissue values.

Tumor histology and gene expression can change with treatment as a result of important changes in cellular processes. We investigated the effects of induction chemotherapy (3 cycles) on *CHD5 *expression in 12 high risk NB cases with available paired diagnostic and post-chemotherapy tumor specimens for qRT-PCR and immunohistochemical analyses. At diagnosis all these tumors (2 locoregional and 10 stage 4 NB) displayed low *CHD5 *mRNA expression and negative immunostaining. Following induction chemotherapy, a significant increase of *CHD5 *transcript and CHD5 positive nuclear staining was detected in 6/12 specimens, together with therapy-induced morphological changes (increased cytoplasm and ganglion-like cell morphology) (Figure 4A and 4C; cases #1-6). All these patients achieved an initial complete or very good response to cytotoxic therapy (chemo- and radiation therapy). At the time of analysis, 5/6 patients were alive with a mean follow-up of 35.62 months (Figure 4B). One case, stage 4 *MYCN *amplified, progressed after a good initial response to chemotherapy and died of refractory bone marrow disease (Figure 4C; case #6). Bone marrow aspirate smears of this patient exhibited widespread tumor dissemination with CHD5 negative neuroblast aggregates (data not shown).

In contrast, low gene and protein expression levels persisted in the 6 remaining post-therapy specimens (6 stage 4 NB; 3/6 *MYCN *amplified and 1p36 deleted tumors) (Figure 4A and 4C; cases #7-12). Therapy induced neuroblastic differentiation was observed in only one of these samples (case #7), a stage 4 NB with aberrant morphological changes. All 6 patients died of rapid disease progression with no signs of clinical response; with a mean survival of 12.73 months (Figure 4B).

These observations suggest a relationship between CHD5 expression reactivation and response to induction therapy and subsequent patient outcome.

## Discussion

Gene expression of CHD5, an ATP-dependent chromatin remodeling enzyme, has been reported to be restricted essentially to the nervous system [8,10]. We describe for the first time that CHD5 is a neuron specific protein in normal neural tissue, with variable immunostaining intensity and intracellular localization among the neuron types of the cerebral cortex. Recent evidences suggest that the diverse neuron cell classes derive from distinct embryonal germinal zones and are characterized by specific cell signaling systems that regulate neural stem cells throughout the developing brain [13-15]. Thus, neuronal cells adopt a brain layer fate determined by their molecular profiles [14]. While we did not observe a layer specific distribution of CHD5 in the cerebral cortex, we did note an association of CHD5 expression with neurons with distinct morphological, physiological and neurochemical features.

In normal neural tissue, glial cells appeared consistently devoid of *CHD5* expression. In human glial tumors, chromosome arm 1p allelic loss is a frequent genetic abnormality, especially in oligodendrogliomas (70-85%) and astrocytomas (20-30%) [16]. Recently, low levels of *CHD5* expression have been reported in gliomas with 1p deletion, whereas nondeleted tumors displayed expression levels comparable to normal brain [4]. Thus, deletion of CHD5 has been proposed as an initiating event in gliomas [4]. Our findings, however, suggest that the role of CHD5 as a tumor suppressor in glial tumors needs further investigation.

NTs are embryonal cancers that are assumed to originate from primitive sympathetic neuroblast aggregates located in neural crest derived sympathetic nervous system. We observed how primitive neuroblast aggregates found in fetal adrenal gland specimens generally lack CHD5 expression. Interestingly, only a few cells were found with a variable degree of nuclear reactivity in larger aggregates. To date, the fate of these immature neuroblastic aggregates remains unsolved, and spontaneous involution and cell maturation have been proposed [17]. The immunoreactivity observed in a small proportion of neuroblasts within these islets could suggest the establishment of CHD5 expression prior to their disappearance; however, no evident differentiating features were observed in these immunopositive cells that suggested the activation of the maturation process.

In NTs, CHD5 is essentially expressed in the nucleus of differentiating neuroblastic cells and ganglion cells, and absent in the Schwannian stromal component. However, the most intense immunoreactivity was observed in stage 4s NB, a rare subgroup of histologically undifferentiated, highly proliferative, metastatic tumors with a high incidence of spontaneous regression, affecting young infants. Accurate distinction of spontaneously regressing infant NB from high risk infant stage 4 can be difficult, but critical for therapeutic decisions. In our hands, the intensely positive CHD5 nuclear staining enabled a clear distinction of stage 4s NB from stage 4 NB, which was consistently immunonegative. These results are consistent with our previous gene expression profiling study, where similar differential *CHD5 *expression profiles were observed amongst infants with disseminated NB subgroups [18]. Thus, CHD5 immunohistochemical staining may be clinically useful for a more accurate characterization of disseminated infant NB.

In NB, CHD5 nuclear staining was strongly associated with established favorable prognostic variables like low clinical stage, age at diagnosis <12 months and favorable histology. Our findings suggest that CHD5 protein expression may accurately define NB risk groups and may, therefore, be a prognostic marker. Evidence is provided by the statistically significant association found between high CHD5 immunoreactivity and favorable OS and EFS. These results are consistent with recent studies reporting a strong association of *CHD5 *mRNA levels with patient outcome in NB [5,10]. Furthermore, Cox multivariate analyses suggest that the prognostic value of CHD5 protein expression is independent of other clinical and biological variables currently used in risk stratification of NB patients and could therefore represent an immunohistochemical marker of prognosis in NB.

Currently, risk stratification of NB patients is performed by combining different markers with strong prognostic impact, including patients' age at diagnosis, tumor stage, genomic amplification of the oncogene *MYCN*, copy number alterations of chromosomal regions 1p, 11q and 17q, tumor DNA content [1,19] and Shimada histological score [20]. However, despite elaborate risk stratification strategies, outcome prediction in neuroblastoma is still deficient. In recent years, to improve risk assessment additional prognostic indicators such as gene-expression signatures [21-23], combined genomic and molecular signatures [24] or expression levels of single candidate genes, e.g., *Trk *(NTRK) family of neurotrophin receptors [25,26], *FYN *[27], *PRAME *[28] and *ZNF423 *[29], have been associated with NB clinical behavior. Expression of the Trk family receptors has been the most extensively characterized marker in NB and has been found to be consistently correlated with the biology and clinical behavior of NB. Based on our results, there is an apparent similarity between the expression patterns of *CHD5 *and *TRKA *in NB and their patterns of association with NB disease outcome. TRKA expression has been reported to be high in biologically favorable NB tumors and inversely associated with *MYCN *amplification [30]. The prognostic value of the immunohistochemical detection of TrkA has also been examined and reported to be high, especially in combination with Ha-Ras expression pattern [31,32]. Further IHC studies have correlated the lack of TrkA expression with metastatic malignant NB [33]. However, in the latter study, 34% of the patients with stage 4 NB displayed TrkA expression, a subset of which died of aggressive metastatic disease despite TrkA expression [33,34]. In our study, the majority of stage 4 NB either lacked CHD5 immunoreactivity (83%) or exhibited weak nuclear staining (13%), a high risk phenotype according to our scoring system. Only one stage 4 tumor was found to be clearly immunoreactive for CHD5; at the time of analysis the patient is alive, 29 months from diagnosis. These observations further confirm CHD5 as a powerful prognostic marker that could complement other known markers such as age at diagnosis, stage, *MYCN *status, cellular DNA content, 1p deletion and tumor histology. However, the potential clinical use of this marker must be tested in larger, prospective cohorts.

It is known that tumor histology and gene expression can change with treatment as a result of important changes in cellular processes, e.g., induced tumor differentiation, DNA repair, apoptosis and tissue necrosis. Undifferentiated NB occasionally exhibit neuroblastic maturation in response to chemotherapy. Assessment of *CHD5 *gene and protein expression in NB post-therapy specimens revealed that tumors with evident neuroblastic maturation showed both *CHD5 *gene and protein reactivation. Notably, none of these tumors harbored 1p deletion. Conversely, in tumors where minimal or no morphological changes were observed in the post-treatment specimens, low *CHD5 *expression persisted. These observations suggest the existence of a subset of tumors within high risk NB where *CHD5 *expression can be reactivated from the silenced state by standard chemotherapy. Remarkably, when post-therapy reactivation was observed, *CHD5 *expression was largely associated with disease response to cytotoxic induction therapy and subsequently with longer patient OS. All 12 patients included in the study received the same treatment, nevertheless some tumors failed to respond. At present, treatment response in NB is routinely evaluated by monitoring urine levels of catecholamine and its metabolites (VMA/HVA ratio) and by estimating the decrease in the size of measurable lesions with conventional imaging modalities, such as computed tomography (CT) or magnetic resonance imaging (MRI). At the time of second-look surgery, the degree of induced tumor cell differentiation and the extent of necrosis can also be useful to estimate treatment response. However, no biological markers for tumor chemotherapy responsiveness have been reported in NB. The use of such biomarkers would make chemotherapy more effective for individual patients by allowing timely changes of therapy in the case of nonresponding tumors. Furthermore, markers reflecting tumor response can function as surrogates of long-term outcome. Taking into account the small cohort of cases that may have led to an overestimation of the data, our findings would suggest that restoration of CHD5 expression could be a surrogate marker of treatment response that can be clinically useful to identify patients that do not benefit from conventional treatment. These results warrant further investigation in a larger cohort of uniformly treated patients.

In summary, we report that the differential expression of the neuron-specific protein CHD5 accurately defines NB risk groups and may represent a marker of outcome in neuroblastoma that can be tested by conventional immunohistochemistry. In high risk NB patients, re-establishment of CHD5 expression following chemotherapy should be tested prospectively as a surrogate marker of treatment response.

## Competing interests

The authors declare that they have no competing interests.

## Authors' contributions

CL, IG and JM are responsible for the initial conception and overall hypothesis of this study. IG, GM and CL are responsible for the design of this manuscript, including the original draft and subsequent revisions. IG, GM, ER, MS, TG, JR, NKC, CdT, JM MK, RG, AAP and CL were involved with the interpretation of data, draft and revision of this manuscript. CdT provided guidance for many of the experiments. NKC, TG, MK, RG and AAP are responsible for the procurement and cryopreservation of NBT tissue specimens derived from MSKCC and CHB/DFCI. ER, IG, GM, JM and CL were responsible for the procurement and cryopreservation of NBT tissue specimens derived from the Spanish institutions. ER, IG GM, CL and MS are responsible of inmunohistochemical analyses. MS evaluated tumour specimens for staging classification, tumour content. JM and CL are responsible for patient clinico-biological database management. CC, GM, PG and ER are responsible for the *in vivo *study. IG, GM and CL are responsible for the quantitative PCR experiments and inmunoblotting. JR and CL are responsible of statistical analyses. All authors were involved in the drafting and revisions for this manuscript. All authors read and approved the final manuscript.

## Supplementary Material

Additonal file 1 **Clinical and biological characteristics of 90 NT specimens included in the study**. INSS = International Neuroblastoma Staging System; Diagnosis: NB = neuroblastoma, GNB = ganglioneuroblastoma, GN = ganglioneuroma; Disease status: A = alive, D = dead; EFS = Event free survival; qRT-PCR = Quantitative real-time PCR; IHC = immunohistochemistry; n.a = not available data **Immunohistochemical analysis of CHD5 expression in NTs **Results are displayed as percentage of CHD5 immunopositive cells present in each tumor specimen. Staining intensity: 0 = negative; 1 = weak staining; 2 = strong staining, 3 = very intense staining. Proportion of positive cells values (<25% = 1; 25-75 = 2; >75% = 3). Intensity and positive cell values were multiplied together to provide a single score for each case. Click here for file

Additonal file 2 **A. Immunohistochemical staining of FFPE sections of two immunopositive neuroblastic tumors using two different batches of the anti-CHD5 antibody (T00251-A1 and T00251-A02); B. Immunochemical assay with the anti-CHD5 antibody (Strategic Diagnostics, DE) on mouse xenografts derived from human NB cell lines**. The specificity of the anti-CHD5 antibody was validated by immunohistochemical assays on FFPE sections of mouse xenografts of human NB cell lines (SK-N-JD, SK-N-LP and SK-N-AS). In these NB cell lines *CHD5 *gene expression is very low or absent (data not shown), similar to previously reported data (ref. 5, ref. 10). Two different anti-CHD5 antibody batches (T00251-A1 and T00251-A02, Strategic Diagnostics, DE) were tested. Ganglioneuroblastoma FFPE tissue sections were used as positive control samples. All the analyzed xenographs were composed nearly exclusively (>95%) of neuroblastic cells exhibiting no CHD5 nuclear staining and faint cytoplasmic staining (when present). Only few (<5%) immunopositve cells were observed in the SK-N-LP xenograft. However, viable tumor cells in the SK-N-LP xenograft where negative for CHD5 nuclear staining, similar to SK-N-JD and SK-N-AS. These results were comparable to the immunostaining pattern observed in undifferentiated high risk NB tumors. The GNB ganglionar cells showed intense nuclear and diffused cytoplasm immunostaining. Click here for file

Additional file 3 ***CHD5 *mRNA expression levels in NTs **Results are displayed as mean expression levels of NT subgroups obtained from two independent analyses. HR = high risk NB (Stage 4 and Stage 3 *MYCN *amplified); LR = low risk NB (stage 1, stage 2 and stage 3 *MYCN *non-amplified); GNB= ganglioneuroblastoma; GN = ganglioneuroma. Quantification was performed relative to normal fetal brain. Error bars illustrate the variability amongst the samples of each NT subgroup. Click here for file

Additional file 4 **Comparison of *CHD5 *mRNA and protein expression**. The lineal graph shows **c**omparison between *CHD5 *mRNA levels and protein immunoreactivity in 34 NB cases. Low CHD5 protein scores were associated with lower mRNA levels (negative z-score values), and high IHC scores with high mRNA expression (positive z-score values), (Spearman's correlation analysis rho = 0.774; P < 0.001). Low risk tumors, stage 4s and infant stage 1 NB tumors showed very intense nuclear staining in comparison to the observed transcript levels (cases # 1-6, 30, 31 and 33). Click here for file

Additional file 5 **Cox multivariate análisis**. Cox multivariate regression analysis has been performed using clinical and biological variables currently used in risk stratification of NB patients (INSS stage, age at diagnosis, *MYCN *status and 1p LOH) in combination with the CHD5 IHC. The analysis has been performed sequentially, adding one variable at each step, in order to assess how the presence of each variable influences the performance of CHD5. CHD5 IHC remained statistically significantly associated with overall survival in all the analyses, except when the 1p LOH parameter is included in the overall survival analysis. This is due to the strong association of the expression of *CHD5*, located on 1p36, with chromosome 1p status. All the rest of variables, except for *MYCN *amplification, were not statistically significant. For event free survival analysis, CHD5 IHC is the only variable that remained statistically significant along the whole analysis, even in the presence of 1p LOH. IHC = Immunohistochemical analysis; INSS = International Neuroblastoma Staging System; HR = hazard ratio; CI = confidence interval. P-values are two sided. Click here for file
