# Supplementary material for: Expression of the neuron-specific protein CHD5 is an independent marker of outcome in neuroblastoma
Source: Mol Cancer. 2010 Oct 15;9:277. doi: 10.1186/1476-4598-9-277 (PMC2992029; doi:10.1186/1476-4598-9-277)
Supplement: Additonal file 2 — A. Immunohistochemical staining of FFPE sections of two immunopositive neuroblastic tumors using two different batches of the anti-CHD5 antibody (T00251-A1 and T00251-A02); B. Immunochemical assay with the anti-CHD5 antibody (Strategic Diagnostics, DE) on mouse xenografts derived from human NB cell lines. The specificity of the anti-CHD5 antibody was validated by immunohistochemical assays on FFPE sections of mouse xenografts of human NB cell lines (SK-N-JD, SK-N-LP and SK-N-AS). In these NB cell lines CHD5 gene expression is very low or absent (data not shown), similar to previously reported data (ref. 5, ref. 10). Two different anti-CHD5 antibody batches (T00251-A1 and T00251-A02, Strategic Diagnostics, DE) were tested. Ganglioneuroblastoma FFPE tissue sections were used as positive control samples. All the analyzed xenographs were composed nearly exclusively (>95%) of neuroblastic cells exhibiting no CHD5 nuclear staining and faint cytoplasmic staining (when present). Only few (<5%) immunopositve cells were observed in the SK-N-LP xenograft. However, viable tumor cells in the SK-N-LP xenograft where negative for CHD5 nuclear staining, similar to SK-N-JD and SK-N-AS. These results were comparable to the immunostaining pattern observed in undifferentiated high risk NB tumors. The GNB ganglionar cells showed intense nuclear and diffused cytoplasm immunostaining. [file 1476-4598-9-277-S2.doc]

**Additional File 2.**

**A.** Example of immunohistochemical staining of FFPE sections of two immunopositive neuroblastic tumors using two different batches of the anti-CHD5 antibody (T00251-A1 and T00251-A02).


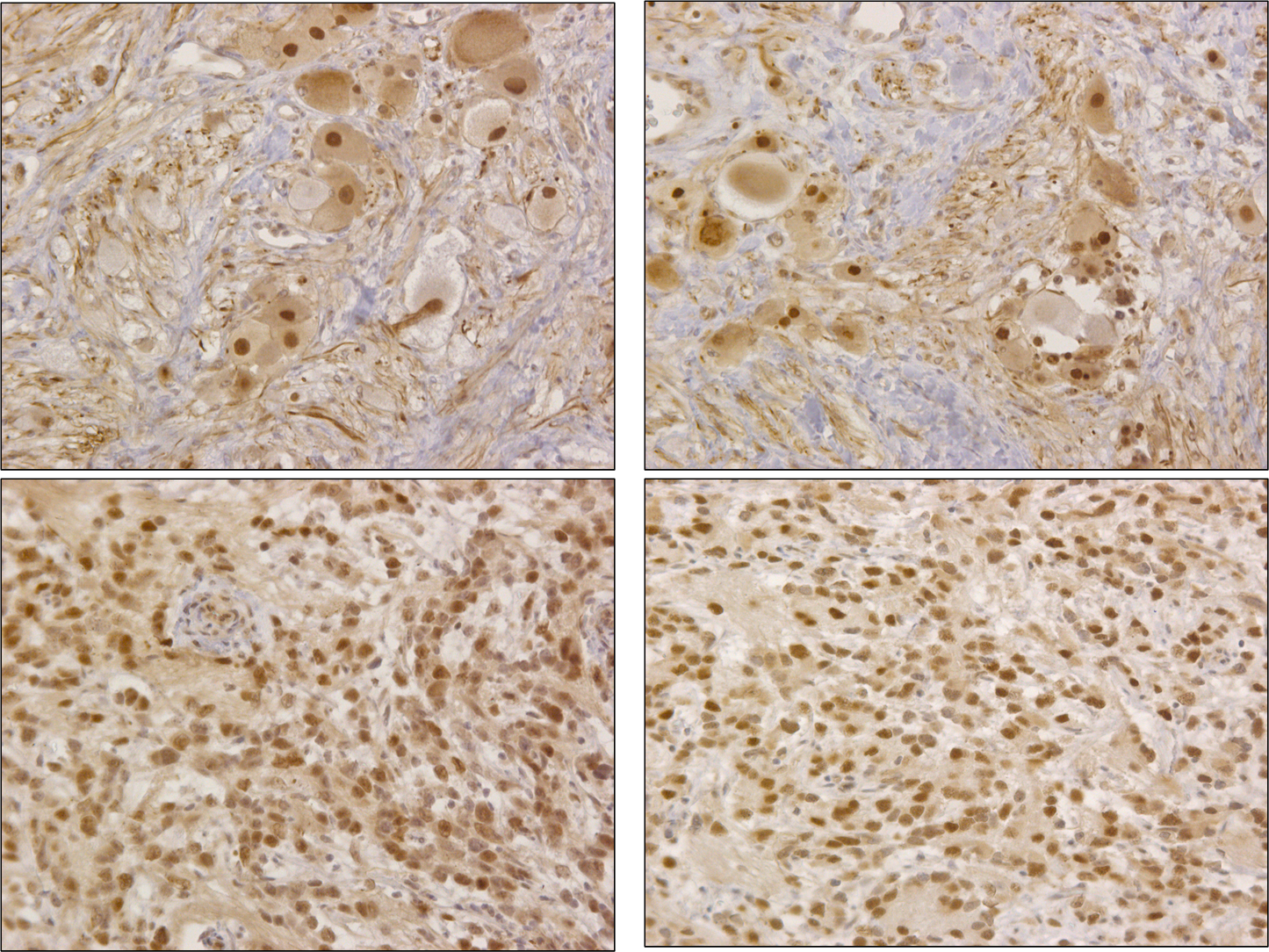


**GNB**

**SD Ab. T00251-A1**

**SD Ab. T00251-A02**

**Loco-regional NB**

**B.** Immunochemical assay with the anti-CHD5 antibody (Strategic Diagnostics, DE) on mouse xenografts derived from human NB cell lines.


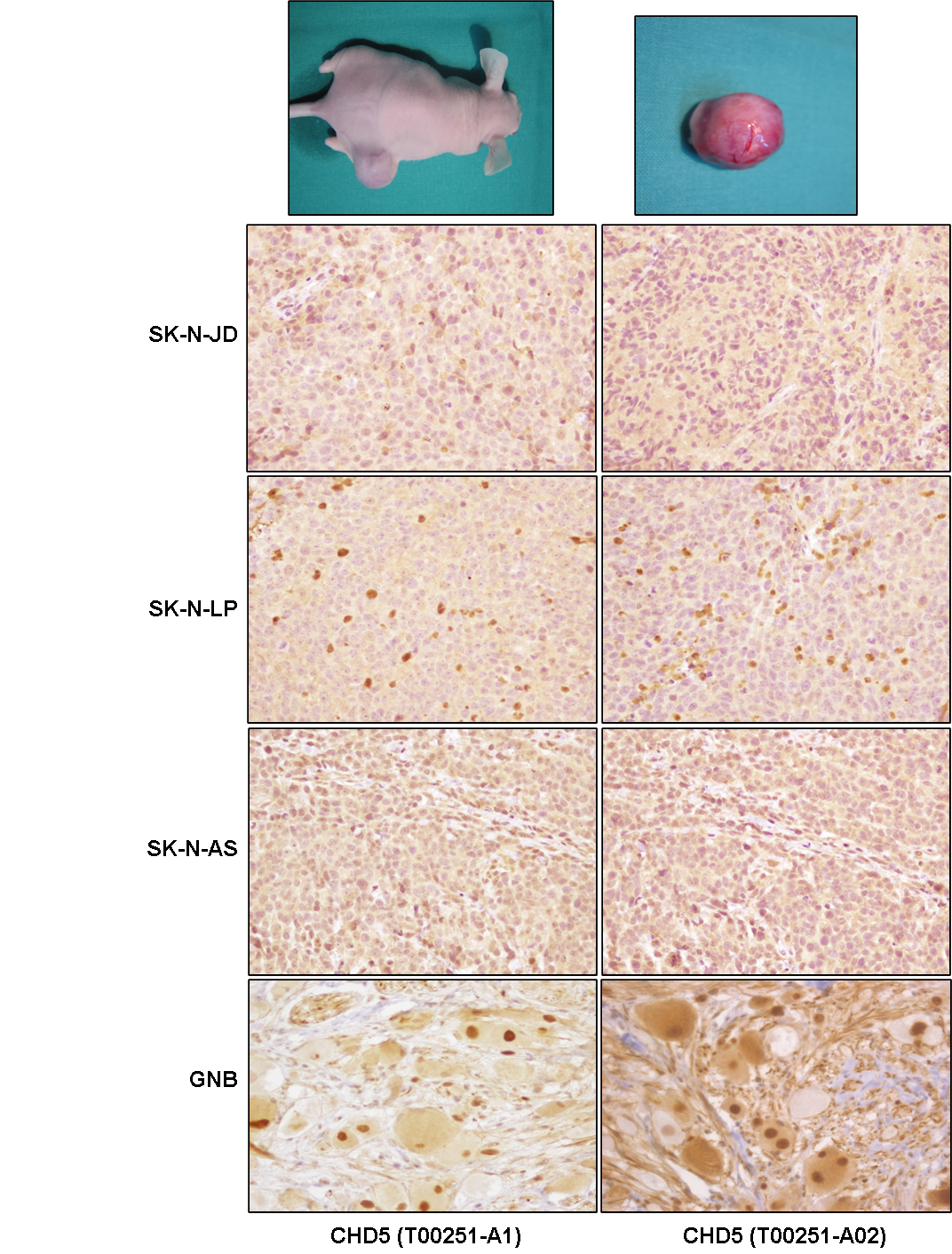


The specificity of the anti-CHD5 antibody was validated by immunohistochemical assays on FFPE sections of mouse xenografts of human NB cell lines (SK-N-JD, SK-N-LP and SK-N-AS). In these NB cell lines *CHD5* gene expression is very low or absent (data not shown), similar to previously reported data (5,10). Two different anti-CHD5 antibody batches (T00251-A1 and T00251-A02, Strategic Diagnostics, DE) were tested. Ganglioneuroblastoma FFPE tissue sections were used as positive control samples. All the analyzed xenographs were composed nearly exclusively (>95%) of neuroblastic cells exhibiting no CHD5 nuclear staining and faint cytoplasmic staining (when present). Only few (<5%) immunopositve cells were observed in the SK-N-LP xenograft. However, viable tumor cells in the SK-N-LP xenograft where negative for CHD5 nuclear staining, similar to SK-N-JD and SK-N-AS. These results were comparable to the immunostaining pattern observed in undifferentiated high risk NB tumors. The GNB ganglionar cells showed intense nuclear and diffused cytoplasm immunostaining.
