# Supplementary material for: Expression of the neuron-specific protein CHD5 is an independent marker of outcome in neuroblastoma
Source: Mol Cancer. 2010 Oct 15;9:277. doi: 10.1186/1476-4598-9-277 (PMC2992029; doi:10.1186/1476-4598-9-277)
Supplement: Additional file 4 — Comparison of CHD5 mRNA and protein expression. The lineal graph shows comparison between CHD5 mRNA levels and protein immunoreactivity in 34 NB cases. Low CHD5 protein scores were associated with lower mRNA levels (negative z-score values), and high IHC scores with high mRNA expression (positive z-score values), (Spearman's correlation analysis rho = 0.774; P < 0.001). Low risk tumors, stage 4s and infant stage 1 NB tumors showed very intense nuclear staining in comparison to the observed transcript levels (cases # 1-6, 30, 31 and 33). [file 1476-4598-9-277-S4.doc]

**Additional file 4**. Comparison of *CHD5* mRNA and protein expression.

# = n. patient

INSS = International Neuroblastoma Staging System

*MYCN* status, NA = non amplified; A = amplified
