# Supplementary material for: Expression of the neuron-specific protein CHD5 is an independent marker of outcome in neuroblastoma
Source: Mol Cancer. 2010 Oct 15;9:277. doi: 10.1186/1476-4598-9-277 (PMC2992029; doi:10.1186/1476-4598-9-277)
Supplement: Additional file 5 — Cox multivariate análisis. Cox multivariate regression analysis has been performed using clinical and biological variables currently used in risk stratification of NB patients (INSS stage, age at diagnosis, MYCN status and 1p LOH) in combination with the CHD5 IHC. The analysis has been performed sequentially, adding one variable at each step, in order to assess how the presence of each variable influences the performance of CHD5. CHD5 IHC remained statistically significantly associated with overall survival in all the analyses, except when the 1p LOH parameter is included in the overall survival analysis. This is due to the strong association of the expression of CHD5, located on 1p36, with chromosome 1p status. All the rest of variables, except for MYCN amplification, were not statistically significant. For event free survival analysis, CHD5 IHC is the only variable that remained statistically significant along the whole analysis, even in the presence of 1p LOH. IHC = Immunohistochemical analysis; INSS = International Neuroblastoma Staging System; HR = hazard ratio; CI = confidence interval. P-values are two sided. [file 1476-4598-9-277-S5.doc]

**Additional file 5.** Cox multivariate regression analysis.

| **OVERALL SURVIVAL** | | | |  | **EVENT FREE SURVIVAL** | | |
| --- | --- | --- | --- | --- | --- | --- | --- |
| **Model nº** | **Factor** | **HR and 95%CI** | **p-value** |  | **Factor** | **HR and 95%CI** | **p-value** |
|  |  |  |  |  |  |  |  |
| 1 | *MYCN* | 8.08 (2.43 to 26.93) | 0.0007 |  | *MYCN* | 2.72 (1.07 to 6.88) | 0.0351 |
|  | **CHD5 IHC** | **12.29 (1.56 to 96.49)** | **0.0171** |  | **CHD5 IHC** | **5.97 (1.98 to 17.98)** | **0.0015** |
|  |  |  |  |  |  |  |  |
| 2 | *MYCN* | 6.96 (2.05 to 23.58) | 0.0018 |  | *MYCN* | 2.57 (0.98 to 6.76) | 0.0549 |
|  | **CHD5 IHC** | **9.21 (1.09 to 78.18)** | **0.0418** |  | **CHD5 IHC** | **5.51 (1.69 to 17.95)** | **0.0046** |
|  | Age_12 | 2.13 (0.41 to 11) | 0.3679 |  | Age_12 | 1.22 (0.42 to 3.54) | 0.7132 |
|  |  |  |  |  |  |  |  |
| 3 | *MYCN* | 7.1 (2.03 to 24.84) | 0.0022 |  | *MYCN* | 2.53 (0.96 to 6.7) | 0.0614 |
|  | **CHD5 IHC** | **9.58 (1.07 to 85.89)** | **0.0436** |  | **CHD5 IHC** | **5.17 (1.43 to 18.72)** | **0.0124** |
|  | Age_12 | 2.18 (0.41 to 11.66) | 0.3612 |  | Age_12 | 1.18 (0.4 to 3.53) | 0.7639 |
|  | INSS | 0.91 (0.26 to 3.14) | 0.88 |  | INSS | 1.14 (0.41 to 3.12) | 0.802 |
|  |  |  |  |  |  |  |  |
| 4 | *MYCN* | 13.1 (2.57 to 66.91) | 0.002 |  | *MYCN* | 2.63 (0.81 to 8.58) | 0.1086 |
|  | **CHD5 IHC** | **7.65 (0.79 to 74.4)** | **0.0795** |  | **CHD5 IHC** | **4.17 (1.05 to 16.57)** | **0.0427** |
|  | Age_12 | 2.27 (0.41 to 12.45) | 0.3444 |  | INSS | 1.28 (0.43 to 3.79) | 0.6542 |
|  | INSS | 1.13 (0.31 to 4.1) | 0.8481 |  | Age_12 | 1.3 (0.43 to 3.92) | 0.6435 |
|  | LOH | 0.52 (0.12 to 2.27) | 0.386 |  | LOH | 0.78 (0.27 to 2.22) | 0.6393 |

Cox multivariate regression analysis has been performed using clinical and biological variables currently used in risk stratification of NB patients (INSS stage, age at diagnosis, *MYCN* status and 1p LOH) in combination with the CHD5 IHC. The analysis has been performed sequentially, adding one variable at each step, in order to assess how the presence of each variable influences the performance of CHD5.

CHD5 IHC remained statistically significantly associated with overall survival in all the analyses, except when the 1p LOH parameter is included in the overall survival analysis. This is due to the strong association of the expression of *CHD5*, located on 1p36, with chromosome 1p status. All the rest of variables, except for *MYCN* amplification, were not statistically significant.

For event free survival analysis, CHD5 IHC is the only variable that remained statistically significant along the whole analysis, even in the presence of 1p LOH.

IHC = Immunohistochemical analysis; INSS = International Neuroblastoma Staging System; HR = hazard ratio; CI = confidence interval. P-values are two sided.
